# Work climate from the perspective of nurses: qualitative research

**DOI:** 10.3389/fmed.2023.1199674

**Published:** 2023-07-28

**Authors:** Justyna Kosydar-Bochenek, Sabina Krupa, Tomasz Semań, Wioletta Mędrzycka-Dąbrowska

**Affiliations:** ^1^Institute of Health Sciences, College of Medical Sciences of the University of Rzeszow, Rzeszow, Poland; ^2^Institute of Medical Sciences, Medical College of Rzeszow University, Rzeszów, Poland; ^3^Department of Anesthesiology Nursing and Intensive Care, Faculty of Health Sciences, Medical University of Gdansk, Gdańsk, Poland

**Keywords:** work climate, organizational climate, nurses, qualitative research, feelings of nurses, workplace climate, work environment

## Abstract

**Introduction:**

This study aims to determine the nurses’ view of the work climate. A positive work climate is one of the keys determining factors in improving nurse outcomes and affects patient satisfaction with care.

**Methods:**

In this qualitative research, a semi-structured interview was used to understand nurses’ perceptions of their work environment. The participants’ responses were recorded and transcribed. Between November and December 2021, 22 nurses participated in the study. Purposive sampling was used to choose nurses for the research, and interviews were performed with these nurses utilizing a semi-structured interview form. The interviews were analyzed using a theme analysis.

**Results:**

The themes identified in the data centered on four dominant elements that together shaped the prevailing work climate: participation in making decisions, companionship, job satisfaction, and changes they expect.

**Conclusion:**

It is necessary to implement meetings at the level of departments and hospitals, where employees will receive support from the authorities and learn how they can improve the working climate.

**Implications for nursing management:**

Research findings on the working climate can help hospital managers makers design interventions to create a good working environment for nurses.

## 1. Introduction

The climate is the perception of the work environment by an employee. It’s how employees feel at work, how they are treated, how they evaluate their well-being, and their professional perspectives. A positive work climate is a workplace with trust, cooperation, safety, support, accountability, and equity (1). There are three basic kinds of definitions for the work environment construct in the literature: the first is based on objective and structural aspects of organizations, the second on individual psychological qualities, and the third on both organizational and personal levels (2). This last and most comprehensive approach highlights employees’ perceptions of the structure and processes that occur in work groups while taking into account both organizational and individual points of view (3). Regardless of the description, there is no agreement on the primary components that comprise the constructed work climate. Job satisfaction, organizational commitment, and motivation to continue working (4); ability, recognition, internal organization, satisfaction, information received, management knowledge, and goals and management responsiveness (5); relationship with the boss, work environment, desire for changes, work satisfaction, capacity for making decisions, tolerance, communication and support, the opportunity for advancement, stress (6). A favorable work environment is one of the key concerns of contemporary firms since it encourages increased productivity, contentment, stability, and dedication to the company (7). The role of the work climate in better outcomes for health workers and patients and is evident (2, 8). The creation of a positive work climate is one of the key determining factors to improve healthcare workers’ outcomes and effects on patient satisfaction with care.

The model provided by Perry et al., where a work environment is considered as a portion of the organization primarily formed by individual human behavior and interactions, may be applicable in the particular domain of health services (9). Each work team has its own climate, which is described as the quality of the internal environment that its members feel and which impacts their behavior. It is stated that a change in an organization’s work environment suggests changes in other areas of that organization, such as the efficacy or quality of patient care (2, 10).

A healthy work environment in the healthcare system enables employees to effectively share the shared goal and duty of professional teams, in which each member clearly knows their position and uses their talents and experience to offer better care for their patients (2). Effective workplace climates ensure that workers understand their role within the greater context of the business and what is expected of them. Organizations may better work in this manner to achieve their objectives. An unfavorable work environment may generate substantial stress and personal unhappiness in professionals, which can influence the quality of health care delivered, raise the sense of weariness and discomfort at work, and reduce labor productivity (2, 11, 12).

The purpose of the study reported in this article was to investigate the perceptions of staff nurses about their work climate. This study was conducted to gain an understanding of the nurses’ views of their work environment, the effect of the work climate on nurses and the problems they face, and to develop recommendations for solutions to these problems.

The research questions were as follows:

- “How do nurses perceive the work climate?”
- “Which components of work climate are paid attention to by nurses?”
- “How to improve the working climate in the studied organization?”

## 2. Methods

A qualitative descriptive design was used in the investigation. The interviews for this research were performed using the Consolidated Criteria for Reporting Qualitative Research (COREQ) checklist for interviews (13). The COREQ checklist can be found in File 1. The trustworthiness of this research was ensured using Guba’s four criteria: credibility, transferability, dependability, and confirmability (14).

The study had a qualitative approach and was based on phenomenology, a research technique used to investigate participants’ perceptions and subjective experiences. Phenomenology is the cornerstone of proper nursing research. It values individual perspectives and the interaction between nurses and patients, providing a comprehensive approach to a person. In order to ensure that participants are information-rich, phenomenological research investigations typically employ deliberate sampling techniques (15).

### 2.1. Sample and recruitment

This study was conducted in a 600-bed university hospital in southern Poland. The hospital employs approximately 450 clinical nurses providing acute care for adults. The study involved 25 nurses, of which 22 nurses in the study group met the inclusion criteria and consented to participate in the study. This sample size was compatible with other qualitative investigations and based on recommendations for phenomenological studies. Work experience for a minimum of 12 months and providing informed permission to participate in the research was required for participation in the study. The data was collected between November 22 and December 22, 2021. Prior to participating, each subject was informed about the goal of the research and provided permission. Also guaranteed were anonymity and secrecy. The information was gathered *via* semi-structured interviews. The nature of the interview allowed the interviewers to ask follow-up questions when further information was needed. During a work break, nurses were told about the opportunity to participate in the research. The interviews took place outside of working hours, at a time that was convenient for the respondents. The interviews were taped, transcribed, and returned to the nurses who took part for final approval. The interview lasted between 23 and 30 min. The place of the meeting was the research nurse’s workroom. No one could enter the room at that time, no sounds from outside could be heard.

### 2.2. Questionnaire development

To achieve the study aims, a semi-structured interview was employed. Two researchers conducted a dialogue with nurses using a questionnaire containing nine questions (Table 1). The interview method enabled interviewers to ask follow-up questions when more information was needed. The study team was made up of two professional academic researchers (Ph.D.), both of whom serve as lecturers in a health faculty and have worked as healthcare professionals in the past. The researchers have conducted in-depth interviews before and were able to stay patient and open during each discussion.

**Table 1:** Interview using a questionnaire comprising nine questions.

**Researcher (R). Good morning. Thank you for agreeing to participate in the study. Can you tell us what the climate is like in your workplace?**
Are you involved in patient-related decisions?
Are you informed about administrative decisions made at your workplace?
Are your colleagues willing to cooperate?
Are you proud to work in your unit?
Is your work appreciated in your workplace?
Do you have the resources needed to do your job well?
Do you develop your skills and knowledge in your workplace?
What changes should be made to improve the climate at your workplace?

### 2.3. Data collection

The interviews were done in accordance with the semi-structured interview form developed by the researchers. After obtaining consent from the participants, the interviews were taped using voice recorders, and the interviews were finished on time. Before the interviews, the scope of the study was explained, and it was documented that the permission of the institutions and ethics committees was obtained. The interview records would only be used for research purposes and the interviews were planned to be conducted at a place and on a date co-determined by the nurses and researchers.

At the conclusion of each interview, the researcher summarized the data gathered and asked the participant to express his judgment on the veracity of the information provided. This post-interview procedure allowed participants to provide any additional information to fully explain their comments and avoid any misconceptions.

### 2.4. Data analysis

The sociodemographic data of the nurses were calculated using numbers and mean values. The interviews were listened to multiple times by the researcher and transcribed word for word and then evaluated using the ‘content analysis method’, one of the qualitative data analysis methods. The data were coded by two researchers separately by dividing the data according to their meanings, and codes associated with each other around a certain meaning were combined, and themes were created.

Following coding, two researchers collaborated to compare coding and make the findings relevant by interpreting the data, ensuring coding consistency.

First, depending on the date and time of the interview, each nurse was allocated a code. The study material was then transcribed and examined utilizing phenomenology by the lead researcher. Each respondent was allocated a number based on the order of the interviews. During the qualitative analysis, the allocated numbers were employed. Each number was given the letter ‘P’ for the sake of this article. Personalization has been applied to the responder. To safeguard the subjects’ identity, labels with the letter ‘P’ were utilized.

The theme analysis was carried out using van Manen’s (16) comprehensive, selective, and detail-oriented method. The researcher listened to the audio while reading the transcription, as suggested by the scholar, to discover any contradictions. At least three times, the literal transcriptions were read. The remarks of a responder describing his or her work environment experience were emphasized (selectively). These included thematic remarks about his or her experiences. The researchers used the ‘close-read’ strategy to better comprehend the respondent’s impression of his/her experience, which implies that each phrase or sentence group in each of the interviews was closely scrutinized (line-by-line analysis with a focus on detail). This approach of analysis sought to elicit the core of the event. The narrative reports and interpretive summaries of respondents were coded to assist in identifying the themes arising from the gathered data. The stories of the responders were discovered to be related and consistent. The same themes were found in all categories of responders. Triangulation of the data yielded four integrating common themes: “Participation in making decisions,” “Job satisfaction,” “Companionship/Team collaboration and communication” and “Changes.” Some themes were deleted because they did not fit well with the evolving framework or had poor evidence grounding.

### 2.5. Ethical considerations

This study was approved by the Bioethics Committee of the University of Rzeszow in Poland (KBE No. 2022/013). The authors followed the guidelines of the Declaration of Helsinki (17). To guarantee anonymity, no personal data permitting the identification of the respondents was required. The participants could withdraw from the survey at any moment without providing any justification, and no data was saved.

## 3. Results

The study included 22 nurses, including 19 women and 3 men. The mean age was 38.2. Nurses worked in various hospital departments. The following departments participated in the study: 45.4% from the ICU, 22.7% from rheumatology, 18.1% from neurology, and 13.6% from orthopedics. Years of work in the profession are on average 14.8. Four nurses in the study group did not have any specialization (Table 2).

**Table 2:** Description of study sample.

Category	Number
**Age**	
20–35 years	9
36–45 years	9
Over 46 years old	4
**Sex**	
Male	19
Female	3
**Place of work (unit)**	
ICU	10
Neurology	4
Rheumatology	5
Orthopedics	3
**Years of work**	
Up to 10 years of work	7
From 11 to 20 years of work	11
Over 21 years of work	4
**Specialization**	
Anesthesiology and intensive care	9
Surgical	5
Conservative	4
No	4

The themes identified in the data centered on four dominant elements that together shaped the prevailing work climate: participation in making decisions, companionship, job satisfaction, and changes they expect to improve their work climate, followed by a detailed discussion.

### 3.1. Participation in making decisions

Most of the respondents (18 of 22) say they are overlooked in making decisions. Some people find that their opinion is important in making such decisions:

“We are often asked about our observations during the morning visit. If something happens to the patient, we can suggest to the doctors what we think is right and suggest that a specific drug or infusion be ordered” P6.

“Doctors never ask me what they should do. They often make decisions without consulting us and other doctors” P1.

“I do not feel left out when making decisions. I have a free hand when it comes to, for example, nursing a sick person” P3.

“He never makes his own decisions. If I want to suggest something to the doctor, then only how it is incompatible with what I can do. Of course, some doctors are grateful if I tell them something, but not all of them” P5.

“I carry out the orders of doctors. It rarely happens that they consult me on something. I’m used to it, but it makes me feel a bit worse. Because we should be a member of the team, not only with our hands to work” P21.

“I am often asked for my opinion on patient care. I am involved in making decisions about treatment. At the same time, I know that it depends on the doctor who is on duty” P16.

### 3.2. Companionship/team collaboration and communication

Nurses find that their colleagues are helpful to them. Usually, it is doctors who do not want to cooperate with them. Nurses feel that communication with doctors is difficult and that doctors did not provide them with necessary explanations when sharing a patient’s situation with them.

“My friends often help me with patient care. Even if we do not like each other, I do not think about not helping someone.” P2.

“Every time I ask other nurses or doctors for help, I get it. I have no problem asking for help. Sometimes there are not enough of us on call, but then we unite even more” P4.

“My friends are always understanding of me. When I need to switch duties, there will always be someone willing. We have bad days, but this does not significantly affect the climate in my workplace” P10.

“Nurses stay together, and doctors stay together. We do not have conflicts with our friends, but usually, it is like that with doctors. They treat us as inferior to ourselves. Some people imply that they are better” P8.

“Not all doctors are kind to us. It happens that they shout at us and get irritated. I do not know why they react like that, but other friends always stand behind me if the doctor blames me” P9.

“I believe that the atmosphere is spoiled by the nurse-doctor relationship. At least this is the case in my ward, where doctors do not respect us as their co-workers” P7.

### 3.3. Job satisfaction

The answers varied with job satisfaction. Some people say that satisfaction is related to patient care, but the remuneration and attitude of the authorities negatively affect the perception of work in a positive way.

“I enjoy my work, although we often lose our patients. Now it is getting worse and worse because we have more and more work, and we earn the same. It is frustrating” P14.

“We will never be satisfied with our work until we earn a decent salary and there are no more of us on call. The management does not care about encouraging the employment of nurses” P20.

“I am not satisfied with the work in my department. I’ve never heard praise for my work, we do not get bonuses. It is sad, because if it were otherwise, I would be happy to come to work” P17.

“I like my job and my department, but sometimes it gets frustrated that nobody appreciates us, and we are not rewarded for how hard we work” P12.

“If doctors were more friendly towards us, my satisfaction with work and the work climate would be better. It is a pity that nobody appreciates us because it is very important in our profession” P22.

“I have great satisfaction with my work, but when there are some problems related to the cast, for example, it makes us angry. When arriving for duty, you hope that he will be calm, and it is not always so due to the fact that there are not enough of us” P19.

### 3.4. Changes

The respondents clearly say what changes they expect:

Decision:

“I would like us to be noticed when important decisions are made, especially administrative ones because we are always overlooked” P11.

Contact with supervisor:

“I would like meetings of nurses and doctors to be organized in the presence of superiors (head of the clinic, ward nurse) and that we would be able to explain some situations and not sweep conflicts under the carpet” P13.

“I believe that there is no good flow of information between our superiors and us. We are often the last to learn about some decisions and we often disagree with those decisions” P18.

“Changes are needed, if only because administrative decisions are often made beyond our knowledge and consent. We cannot feel at ease in a place where everyone talks about us, and no one consults with us. We stick together, but the management is not honest with us” P15.

## 4. Discussion

This study evaluated nurses’ views of the work environment and the problems they faced in their work environment. Four main themes, ‘participation in decision making’, ‘companionship’, ‘job satisfaction’, and ‘changes’ that nurses expect to improve their work environment, were determined for the study.

Under the first theme, ‘participation in decision making’, it was determined that most nurses say that they are overlooked when making decisions, which can significantly affect the working climate. Only a few respondents find that doctors consider their opinion when making decisions. In clinical practice, it is necessary for nurses to collect various information needed to care for patients and make clinical decisions with rational and critical reasoning (18, 19). Despite the literature on the decision-making process, to date, complete awareness of the decision-making process of nurses and how it is used in practice remains an area of lively debate (20). The combined use of intuition and evidence-based practices is the best strategy for providing tailored nursing care, which deserves a privileged place in the clinical decision-making process (18).

In the present study, it was found that nurses were not adequately supported by doctors. Nurses find that their co-workers are helpful to them. Our findings indicate that nurses perceive that doctors have trouble cooperating with them.

In Watkins’s study, nurses and physicians generally had different perceptions of nurse-physician collaboration (21, 22). Shared decision-making, teamwork, and communication were recurring themes in reports of perceptions about nurse-physician collaboration. Good communication and collaboration within the healthcare team are critical for generating safe emotional and professional environments for team members and excellent healing (23).

Subsequent studies describe the role of nurses as a supporting role. Nurses are the ones who support patients and their families (24). According to research, it is necessary to create a workplace that will allow nurses to talk freely, rest, and psychological development. It is also necessary to ensure adequate communication in the interdisciplinary group. Relationships in the department should be based on respect, but it is also necessary to consider job satisfaction, which significantly impacts the working environment (25). In our own study, nurses appreciate their work and want to take care of patients as before, but sometimes it is difficult due to the duties related to work, which is sometimes underestimated.

This study states that nurses’ job satisfaction can significantly affect the work climate. In a study by Ayalew, more than half of the nurses had good job satisfaction (26). In our study, nearly half of the respondents were nurses from the intensive care unit. The authors state that nurses working in highly demanding areas, such as intensive care units, are more likely to experience burnout and low job satisfaction than nurses working in less demanding areas (27). Evidence from the review of 11 studies showed that intensive care unit nurses are suffering from moderate to high levels of burnout while experiencing only moderate levels of job satisfaction (28). The impact of job satisfaction on the working climate requires further research. In particular, studies using a qualitative approach that could provide more insight into the study of this compound are insufficient in this area.

According to the research, the primary reason for nurse turnover is job unhappiness in the nursing work environment. Job satisfaction has been linked to high levels of empowerment in nurses (29, 30). The empowerment of nurses and their work satisfaction was shown to have a substantial beneficial relationship. Job happiness is affected differentially by structural and psychological empowerment. Healthcare organizations and management should think about introducing effective interventions to increase nurses’ work satisfaction and decrease turnover (31). This research’s findings emphasize the need for nurse managers to focus on organizational tactics that support workers’ intrinsic motivation. The present nurse shortage and the goal to boost turnover are proving to be a worldwide issue. As a result, nurse managers must devise measures to increase nurse work satisfaction. The successful treatments discovered in this research are the initial step toward developing human resource strategies for healthcare companies. In the study by Niskala et al., the spiritual intelligence training protocol and Professional Identity Development Program were found to be effective in improving job satisfaction (31). Having such training has been beneficial at work and can improve job satisfaction.

Respondents clearly say what changes they expect. They mainly concern organizational and decision-making changes, as well as relationships with other professional groups, mainly doctors. Because of technological improvements, aging populations, changing illness patterns, new discoveries for disease treatment, and political changes and policy efforts, healthcare institutions are continually evolving. Change may be difficult since it contradicts people’s inherent desire for a stable environment, yet it is necessary for progress (32). A survey of healthcare representatives in Sweden discovered that organizational changes in healthcare are more likely to succeed when healthcare personnel can influence the change, feel prepared for the change, and appreciate the value of the change, including recognizing the benefit of the change for patients (33). The organizational atmosphere and organizational commitment have been demonstrated to have a favorable association (34). Another study by Delaney et al. reiterates that good HR management practices, including staff training, are also determinants of a good working climate (35). It is very important to find out what hospital employees think about the working climate. Authors from other centers emphasize that the topic of working climate is very important as it can help to provide patients with high-quality care (36–38).

## 5. Conclusion

The nurses’ work environment is changeable, and very stressful, requiring quick decision-making and action. Ensuring a good work climate and safety in such an environment is an inherently difficult context. Work climate is provided by employees through mutual respect, work, and managers should support their employees in these activities. All healthcare professionals, including nurses, should be able to express their views on dissatisfaction with working conditions. Additionally, superiors and doctors should consider the views of nurses when making decisions related to the functioning of the ward. An important issue is job satisfaction, which significantly affects the willingness to work, professional burnout, and the work environment.

In this paper, you can also see some elements that overlap with what other authors write: no information flow, separation of the group of nurses from doctors, and no possibility of making decisions related to the ward.

In the current epidemic situation, it should be remembered that the work climate is also an indicator that nurses will not leave the profession.

### 5.1. Implications for nursing management

Improving the working climate is possible thanks to the cooperation of the hospital management with the employees of individual departments. It is necessary to create a good working climate wherein nurses will feel safe, healthy, and appreciated by the management, doctors, other staff, and patients. The work climate experienced by nurses depends on many factors, among which deserve special attention: participation in decision-making, ‘companionship/teamwork’, ‘job satisfaction’, and ‘changes’ that nurses expect to improve their work environment. The tasks of the management include monitoring the working climate and its improvement through, among others, applying the principles of fair treatment, good communication, focusing on team integration and cooperation, and improvement of working conditions. In addition, it should be borne in mind that the work climate is an important predictor of job satisfaction and the quality of patient care.

### 5.2. Study limitations

It should be noted that this study has some limitations. As with any qualitative study, the generalizability of the results is limited. This study was conducted in only one study site, no representatives from each hospital ward participated in the study, therefore the results of this study may not be applicable to other hospitals or other hospital wards as they focused on selected wards. Moreover, the data were obtained from nurses’ self-report of their experiences and feelings. The study findings could change by cultural differences. Future studies will also include other hospital wards.

## Data availability statement

The original contributions presented in the study are included in the article/supplementary material, further inquiries can be directed to the corresponding author.

## Author contributions

JK-B: conceptualization. WM-D: formal analysis and supervision. JK-B, SK, TS, and WM-D: methodology and writing – original draft. JK-B, TS, and SK: resources. All authors contributed to the article and approved the submitted version.

## Conflict of interest

The authors declare that the research was conducted in the absence of any commercial or financial relationships that could be construed as a potential conflict of interest.

## Publisher’s note

All claims expressed in this article are solely those of the authors and do not necessarily represent those of their affiliated organizations, or those of the publisher, the editors and the reviewers. Any product that may be evaluated in this article, or claim that may be made by its manufacturer, is not guaranteed or endorsed by the publisher.
